# Sex Differences in the Neural Processing of Aversive Interoceptive Events: The Benefit of Relief

**DOI:** 10.1371/journal.pone.0084044

**Published:** 2013-12-30

**Authors:** Giulia Galli, Akanksha Shukla, Alan N. Simmons, Paul W. Davenport, Martin P. Paulus

**Affiliations:** 1 Department of Psychiatry, University of California San Diego, San Diego, California, United States of America; 2 Veterans Affairs Health Care System, San Diego, California, United States of America; 3 Department of Physiological Sciences, University of Florida, Gainesville, Florida, United States of America; 4 Dipartimento di Scienze Neurologiche e Neurosensoriali, Azienda Ospedaliera Universitaria Senese, Siena, Italy; The University of Queensland, Australia

## Abstract

Do men and women process and experience unpleasant bodily states differently? We used fMRI to determine brain processing before, during and after an aversive respiratory stimulation. No sex difference emerged during anticipation or stimulation. However, after the offset of the stimulation, men but not women showed enhanced activation of brain regions that are important for interoception and reward processing. Moreover, this activation was highest in those males who rated the preceding stimulation as most unpleasant. These results indicate that men are particularly sensitive to reward associated with the termination of an aversive event, which may signal relief.

## Introduction

Interoception refers to the perception of the physiological condition of the body [1]. Individuals are sensitive to a number of interoceptive stimuli, and respiratory sensations are an essential interoceptive experience. Psychophysiological and neuroimaging studies have shown that the sensation of breathing is a complex process which includes a sensory dimension, a strong affective component, and a strong motivational drive to homeostatically regulate the internal state [2], [3], [4], [5]. Affective and motivational processes associated with breathing sensations may begin before a respiratory perturbation sets in [6], and continue after its offset in the form of relief [7].

The insula and the anterior cingulate cortex (ACC) are key brain substrates for the processing of interoceptive states in general [1], [8] and respiratory sensations in particular. However, very little is known about sex-related differences in the engagement of these areas during the processing of interoceptive stimuli. Yet, anecdotal and empirical observations suggest that men and women differ in the way they experience and report bodily states. Women are typically more sensitive to changes in somatic states [9], including breathing [10], [11]. This is also evident from the observation that women have greater sensitivity to experimentally induced pain, with lower thresholds and tolerances to painful stimuli [12]. On the other hand, men appear to have a less sensitive, but more accurate, perception of bodily states [13], [14]. The specific mechanisms that underlie these differences are largely unknown. At the neural level, sex differences in interoceptive processing may be accompanied by differential activations of the insula and the ACC. Evidence of sex differences in the dendritic morphology [15], and functional activation of these areas during emotional and motivational processing [16], [17], [18], hints that this might be the case.

In the present study, we used functional magnetic resonance imaging (fMRI) to examine sex-related differences in the neural processing of an aversive respiratory stimulus. During fMRI, male and female participants responded to the direction of an arrow presented on the computer screen, while they breathed either normally, or against an inspiratory load [19], [20]. This experimental approach has been previously shown to effectively induce a substantial alteration in interoceptive state [2], [5], [6], [21]. One key question to address is at which stage of processing sex differences, if any, arise. Sex differences not only exist in the brain activity linked to the processing of an aversive event, but also in the brain activity preceding it [22]. In addition, extant functional imaging literature has shown specific brain activations after the offset of an interoceptive threat, which has been interpreted as relief [7], [23]. We thus measured neural responses and reaction before, during and immediately after the respiratory aversive stimulation, as well as during normal breathing.

## Methods

### Participants

Thirty volunteers (fourteen women) took part in the experiment. Women and men did not differ in age (women M = 28.6 years, SD = 8.6 years; men M = 28.8 years, SD = 7.9 years; *p* = 0.956), or years of education (women M = 15.7 years, SD = 2.1 years; men M = 15.7 years, SD = 2.2 years; *p* = 0.972). The University of California San Diego (UCSD) Institutional Review Board approved the experimental procedures and each subject provided written informed consent before participating.

### Breathing Apparatus

Volunteers breathed through a mouthpiece with a non-rebreathing valve (2600 series, Hans Rudolph) and wore a nose clip. The apparatus was attached to the scanner head coil in order to avoid mouth muscle contractions. The resistance loads consisted of sintered bronze disks placed in series in a Plexiglas tube, with stoppered ports inserted between disks. Breathing loads were manipulated by removing the stopper and allowing the subject to inspire through the selected port. Based on preliminary data, 40cmH_2_O/L/sec was employed as load.

### Procedure

All subjects were trained to perform the breathing load task before the fMRI session. Each participant was given the following instructions: “This task examines how people feel when breathing becomes difficult. You will breathe through a hose, which makes breathing-in more difficult. It is important for you to know that this test is not physically harmful, but you may feel uncomfortable when you breathe through the hose. You can stop at any time if breathing becomes too difficult. You will be asked to breathe through the hose several times”. Inside the scanner, individuals performed a continuous performance task. Subjects were asked to press a button according to the direction pointed by a black arrow presented centrally on the screen (i.e., subjects had to press the left button if the arrow pointed to the left, and the right button if the arrow pointed to the right). While the arrows were presented, the background colour of the screen served as cue to the impending presentation of the breathing load. Subjects were instructed that a grey background of the screen indicated that no stimulation would be presented. This constituted the baseline condition. Throughout the baseline condition, the arrow was presented on the grey background every three seconds. Subjects were also informed that if the arrow was presented on a yellow background, there was a 25% chance of stimulation. This constituted the anticipation condition, which lasted between six and twelve seconds. Thirty-two anticipation trials were presented in total, followed by stimulation episodes in eight trials. On average, the baseline and anticipation conditions lasted nine seconds, and the post-stimulation twelve seconds. The duration was jittered in time to allow optimal resolution of the hemodynamic response function. Each stimulation episode lasted 40 seconds. The event-related fMRI design consisted of two scans with 256 repetitions (TR = 2 s), yielding a total scan duration of 17 minutes and four seconds. The main behavioural variable was response time during baseline, anticipation, stimulation and post-stimulation. The main neuroimaging-dependent variable was the activation in functionally-constrained regions of interest during anticipation, stimulation and post-stimulation relative to baseline, separately for women and men.

After the end of the scanning session, subjects rated the breathing-load experience on a 10 cm Visual Analogue Scale (VAS), anchored from “not at all” to “extremely”, separately for pleasantness, unpleasantness and intensity dimensions. These ratings served as individual difference measures to correlate the subjective experience of breathing load with brain activation during anticipation, stimulation and post-stimulation.

### fMRI Data Acquisition

Testing was carried out with a 3T GE CXK4 Magnet (General Electric Medical Systems, Milwaukee, Wisconsin) at the UCSD Keck Imaging Center, equipped with 8 high bandwidth receivers that allow for shorter readout times and reduced signal distortions and ventromedial signal dropout. Each session lasted one hour and encompassed a three-plane scout scan (10 s) and a standard anatomical protocol consisting of a sagittally acquired spoiled gradient recalled sequence (field of view = 25 cm; matrix = 192×256, 172 sagittally acquired slices 1-mm thick; repetition time = 8 ms; echo time = 3 ms; flip angle = 12°). An eight-channel brain array coil was used to acquire T2*-weighted echo-planar images (field of view = 23 cm; matrix = 64×64, 40 slices 2.6-mm thick; gap = 1.4 mm; repetition time = 2000 ms, echo time = 32 ms, flip angle = 90°). Rapid image acquisition was obtained via GE’s ASSET scanning, a form of sensitivity encoding (SENSE) which uses parallel imaging reconstruction to allow for sub k-space sampling.

### fMRI Data Analysis

Structural and functional images were processed with the Analysis of Functional Neuroimages (AFNI) software package [24]. EPI images were co-registered using a three-dimensional coregistration algorithm [25] that was developed to minimize the amount of image translation and rotation relative to all other images. Each participant had six motion parameters obtained, three of which were used as regressors to adjust for EPI intensity changes due to motion artefacts. Voxel data points representing outliers relative to surrounding datapoints were eliminated and interpolated. All slices of the EPI scans were temporally aligned following registration to ensure that different relationships with the regressions were not dependent on the acquisition of different slices at different times within the repetition interval.

Regressors of interest were generated to quantify neural activation during anticipation, stimulation and post-stimulation. To this aim, a 0–1 reference function was convolved with a gamma variate function [26] modelling a prototypical hemodynamic response [27] and to account for the temporal dynamics of the hemodynamic response [28]. The convolved time series were normalized and used as regressors of interest. These regressors, along with three motion regressors (roll, pitch, yaw), were entered into the AFNI program 3dDeconvolve to determine the height of each regressor for each subject. The main dependent measure was the voxel-wise percentage signal change (PSC) calculated by dividing the regressor of interest coefficient by the baseline regressor. Spatial smoothing was subsequently applied to the percentage signal change data with a 4-mm-full-width at half maximum spatial filter to account for variations in anatomy for individuals. Data were the transformed into Talairach coordinates based on the anatomical MR image for group-level analyses.

To identify sex and stimulation effects on BOLD activation, PSC during the anticipation, stimulation and post-stimulation was subjected to a linear mixed-effects analysis. We used an implementation of the linear mixed effects model in the R statistical package (http://cran.us.r-project.org/) which estimates the parameters of the mixed model using Maximum Likelihood Estimation (MLE). Sex of participant (men, women) and stimulation (anticipation, stimulation, post-stimulation) were used as fixed factors, and subject was used as random factor. In order to guard against Type I error, voxel-wise statistics were calculated using the AFNI program Alphasim, which estimates statistical significance based on Monte-Carlo stimulations. Based on the Alphasim program, it was determined that, given the spatial smoothing of 4 mm FWHM and a voxel-wise p<0.01, the volume threshold for clusterwise probability of 0.05 for the insula was 256 uL, for the ACC was 256 uL, and for the MPFC was 320 uL. Only clusters meeting these criteria were considered for further analysis. A constrained region of interest (ROI) analysis approach was used on brain regions implicated in emotion processing. Stereotactic coordinates of these ROIs were based on standardized locations taken from the Talairach atlas [29].

## Results

### VAS Scales

Mean VAS scores were submitted to a mixed-model ANOVA with dimension (pleasantness, unpleasantness and intensity) as within-subjects factor and a between-subjects factor of sex. There was neither significant main effect of sex (*p* = 0.485), nor a significant interaction between sex and dimension (*p* = 0.418), indicating that men and women did not differ in their subjective experience of the aversive stimulation.

### Task Performance

The latency to respond to the continuous performance task was submitted to a mixed-model ANOVA with a within-subjects factor of stimulation (normal breathing, anticipation, stimulation, post-stimulation) and a between-subjects factor of sex (men, women). The main effect of stimulation was significant (Greenhouse-Geisser corrected, *F*_(1.3, 34.6)_ = 17.27, *p*<0.001). Response times were slower during post-stimulation compared to normal breathing, anticipation and stimulation (Bonferroni corrected pairwise comparisons, all *p*s<0.001), which in turn did not differ from one another (*p*s>0.358). No interaction with biological sex emerged (*p* = 0.198). Accuracy of the continuous performance task was close to ceiling and therefore was not considered.

### Neuroimaging Results

**Figure 1:**
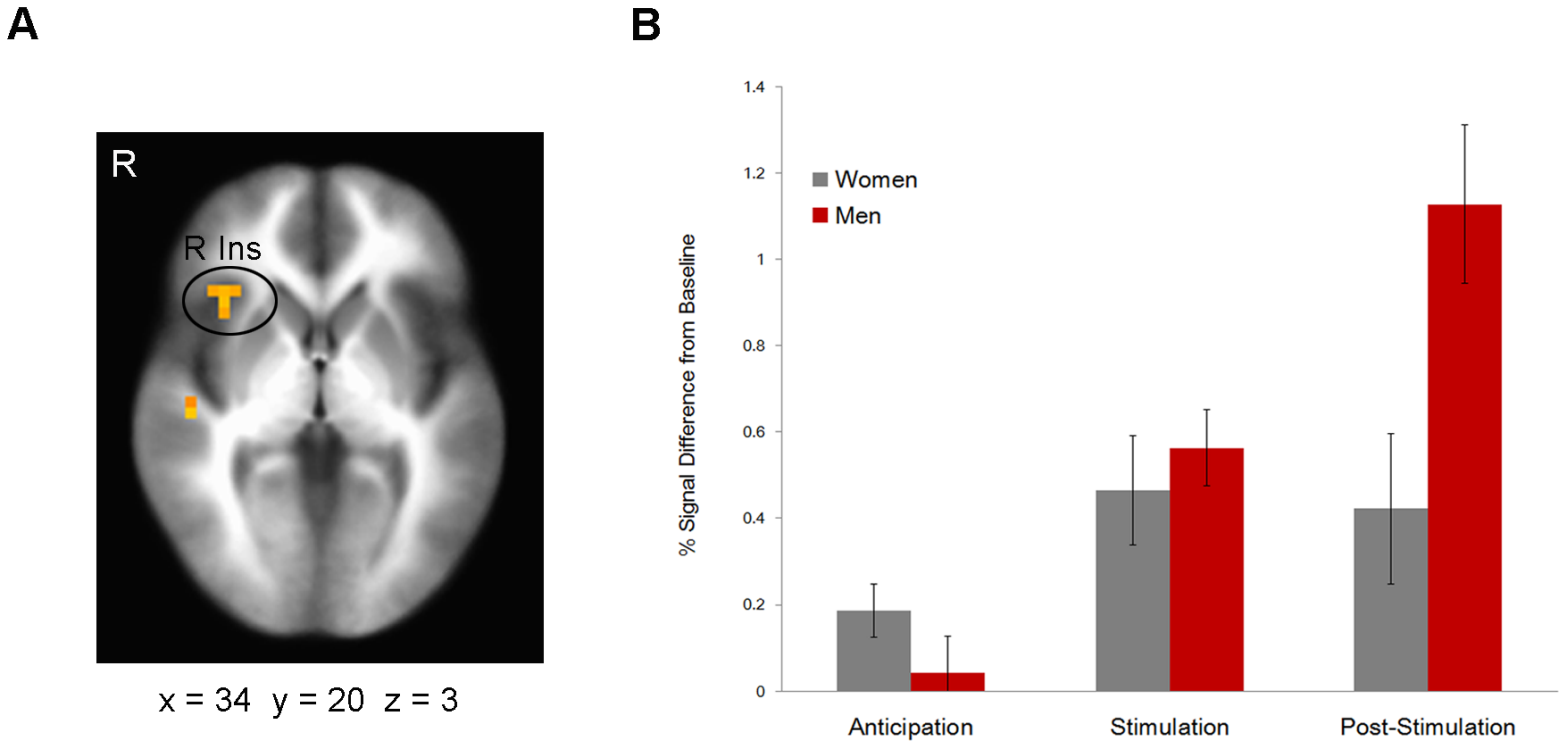
Sex differences in the processing of interoceptive events. (**A**) The right insula is differentially activated in men and women. (**B**) This difference is driven by higher activation in men after the aversive stimulation (error bars represent standard error).

**Figure 2:**
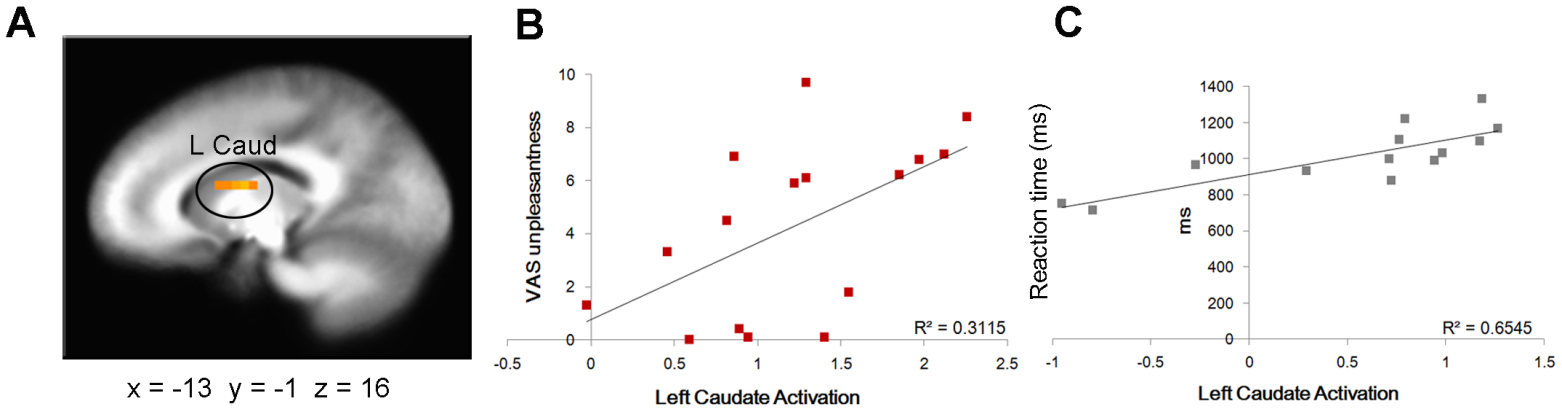
Correlations between brain activations, self-rating of the breathing load and task performance. (**A**) Activation of the left caudate after the aversive stimulation is correlated with the unpleasantness of the stimulation in men (**B**) and with RTs during the continuous performance task in women (**C**).

The main effect of task, irrespective of gender and stimulation (anticipation, stimulation, and post-stimulation), produced activations in a number of brain regions involved in respiratory sensations such as bilateral insula and anterior cingulate cortex (Figure S1). The linear mixed effects models revealed a significant interaction between sex and stimulation in the right insula, right ACC and bilateral caudate (Table 1). As evident from Figure 1 (panels A and B), the interaction resulted from enhanced activation after the aversive stimulation in men, but not in women. This pattern was consistent across regions. Percentage signal change before or during the stimulation did not differ between men and women. To help discern the functional role of post-stimulation brain activity, percentage signal change values after the stimulation in each region reported in Table 1 were correlated with RTs during the continuous performance test, and with the VAS scores. Pearson’s correlations were performed separately for men and women. As shown in Figure 2 (panels A and B), increased post-stimulation activation of the left caudate was associated with higher unpleasantness ratings of the stimulation (*r* = 0.56, *p* = 0.025) in men. Post-stimulation activations of the other brain regions reported in Table 1 did not correlate with VAS scores in men. No significant correlations with VAS scales emerged in women. Instead, in women brain activations after the stimulation correlated with RTs. Significant positive correlations were found in the right posterior (*r* = 0.58, *p* = 0.038) and anterior (*r* = 0.68, *p* = 0.010) insula, left (*r* = 0.81, *p* = 0.001) and right (*r* = 0.71, *p* = 0.006) caudate (Figure 2, panels A and C). No significant correlations with RTs emerged in men.

**Table 1:** Brain regions that show a significant interaction between sex and stimulation.

Anatomic Region	BA	Volume (µL)	x	y	z	F
Right Posterior Insula		576	43	–18	14	9.45
Right AnteriorCingulate	32	448	15	39	–3	7.05
Left Caudate		448	–13	–1	16	5.90
Right Anterior Insula		384	34	20	3	7.53
Right Caudate		320	11	2	17	5.43

## Discussion

The present study examined sex-related differences in the neural correlates of aversive interoceptive stimulation. Prior studies have shown reliable sex-related differences during the anticipation and processing of emotional exteroceptive stimuli [22], [30], [31]. This investigation provides strong, yet unexpected, evidence that sex differences only arise in the neural activity that follows the termination of an aversive interoceptive stimulus. These differences were evident in the ACC, bilateral insula and caudate, which have been implicated in interoceptive and reward processing. Importantly, men, but not women, who reported the highest perceived unpleasantness in response to the breathing load also showed the strongest activation in reward-related processing areas. Thus, one way to interpret these findings is that the termination of a highly aversive interoceptive stimulus generates activation in reward processing areas, which might signal relief in men but not in women.

Previous studies have shown activations of the insula during the subjective experience of breathing load [6], [32], [33], [34], [35], [36], and other interoceptive tasks such as heartbeat and pain perception [1]. In particular, the anterior insula appears to be primarily related to the subjective experience of interoceptive stimulation, and the posterior insula to its objective intensity [37], [38]. Notably, growing evidence suggests that the insula plays a role not only during the processing of an aversive stimulation, but also during its anticipation [6], [39], [40]. As typically observed [33], [34], [36], we found that the insula was jointly activated with the ACC. The ACC is involved in a number of cognitive and emotional processes [41]. It has been suggested that the insular cortex and the ACC form part of a network in which the insula represents the subjective experience of internal states and the ACC the initiation and control of goal-directed behaviour [1].

The enhanced activation of the interoceptive network after the stimulation in men may reflect affective or motivational processes that come into play once the aversive stimulation has ceased. One likely candidate in this respect may be relief. Relief is a subjective experience that can be experienced in relation to a number of interoceptive states. Relief from pain and thirst is associated with brain activation of the ACC [42], [43], bilateral insula [43], and the caudate [44], the same regions that are differentially activated by men and women in the present study. The neural correlates of relief from a breathing load have been investigated in one study [5], which demonstrated that relief was associated with enhanced activation in several subregions of the left ACC and the right caudate. Interestingly, that study included only male volunteers.

Previous studies have shown that the emotional process following the termination of a breathing load does not only involve the experience of decreased intensity of the aversive stimulation, but also a positively-valenced process [7], [45], which has been labelled as “respiratory euphoria” [46]. In other words, it feels good to no longer have to work hard to breathe. Thus, relief from load or “respiratory euphoria” may be considered a dominant motivational drive or a type of intrinsic reward, which is consistent with the similarities of the brain response to relief and to appetitive reward [43]. Furthermore, brain-behaviour correlations in the present study support the link between relief and reward. In men, the more unpleasant the aversive stimulation was experienced, the largest the activation after the stimulation of the left caudate, an area implicated in reward processing [47]. This is in line with the results of a recent psychophysical study [23], which has shown that the magnitude of relief increases with the intensity of the preceding aversive stimulation. No correlation emerged between VAS scores and brain activation after the stimulation in women. Brain activation in women instead correlated with response times during the continuous performance task. This suggests that brain activity after the aversive stimulation in women may reflect the detection of a change in internal state, whereas in men the termination of an aversive interoceptive stimulus acts as a motivational process.

The question arises as to why relief would be experienced to a greater degree in men compared to women. One hypothesis is that men are more sensitive than women to this type of hedonic reward. This fits with data showing that men are more sensitive to monetary and personal rewards [48], [49]. The link between sex differences, relief and reward raises the intriguing possibility that some male-predominant personality traits, such as sensation seeking [50], [51], could at least partly be due to the pleasure associated with relief. On this account, it is worth noting that the rewarding aspect of relief has previously been used to explain a variety of paradoxical behaviours, such as sky diving [52]. One application of this finding would be the gender differences in processing reward related to drugs of abuse. It has been suggested that the motivational basis of substance abuse is the reduction of aversive internal states, such as withdrawal symptoms [53]. In men, this state of relief may be one mechanism that enhances the likelihood of future substance intake, or the transition from initial drug use to drug addiction.

Interestingly, in both sexes response times at the continuous performance task were significantly slower after the offset of the stimulation compared to the stimulation itself or its anticipation. Previous studies on pain have demonstrated a detrimental effect of aversive interoceptive stimulation on attentional performance [54], suggesting that painful stimuli limit the availability of cognitive resources. Our findings suggest that the termination of the aversive stimulation may be even more salient in capturing attention.

The present study did not set in to investigate the neural processing of relief per se, but rather the neural activity associated with different stages of interoceptive processing. Our design thus did not include a direct manipulation or measurement of relief. As a consequence, the interpretation of our data in terms of relief and reward is post-hoc and some caution is warranted in embracing this view. For instance, it is possible that the relatively slow time course of the hemodynamic response captured stimulus- rather than post-stimulus-related processing, or a mix of the two. The current design could be improved by including subjective ratings of relief, and measuring how brain activations vary as a function of the intensity and pleasantness of relief. This would be crucial to attribute any post-stimulus brain activation to the subjective feeling of relief and reward-related personality traits. Another limitation of the present study is that we cannot rule out effects of intervening variables such as smoking status, anxiety or depression levels, and baseline pulmonary function. A number of studies have demonstrated sex-related differences in the prevalence of affective disorders [55] and respiratory function, including different breathing patterns during resistive loads [9], [56]. However, there is little reason to expect any such difference to selectively affect the neural activity after the breathing load has ceased.

In summary, the present study has shown that men and women engage different neural networks when confronted with an interoceptive threat. Crucially, these differences are related to mechanisms set in train after the interoceptive threat has been removed, rather than to the actual processing of threat. This may signal relief and may help understand why men and women respond and behave differently to aversive interoceptive states.

## Supporting Information

Figure S1 **Brain activations irrespective of task, collapsed across men and women.**
(DOCX)
