# Supplementary figures and images for: Sex Differences in the Neural Processing of Aversive Interoceptive Events: The Benefit of Relief
Source: PLoS One. 2013 Dec 30;8(12):e84044. doi: 10.1371/journal.pone.0084044 (PMC3875519; doi:10.1371/journal.pone.0084044)

**Figure S1**: Main effect of experimental manipulation


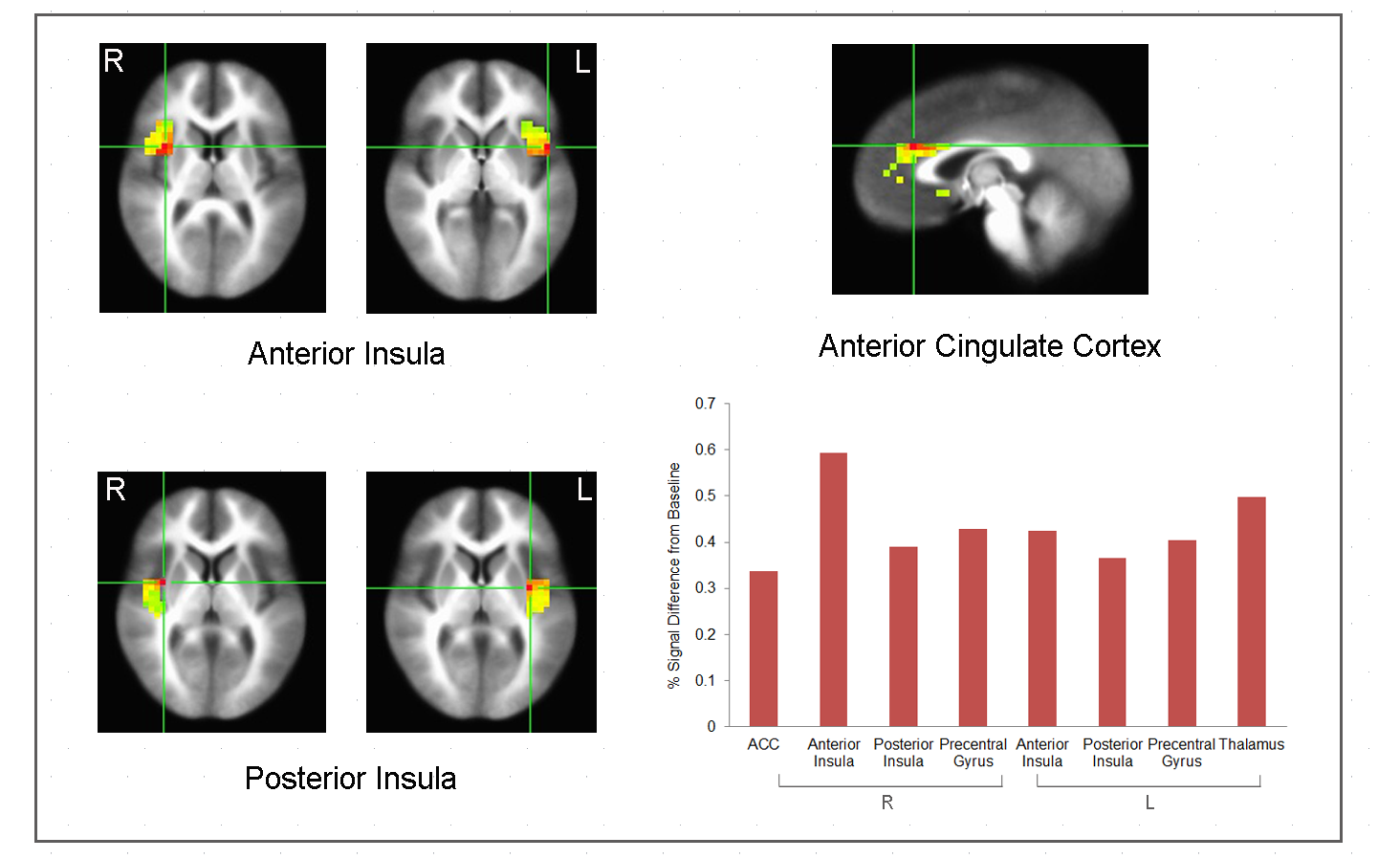

Supplement: Figure S1 — Brain activations irrespective of task, collapsed across men and women. (DOCX) [file pone.0084044.s001.docx]
